# Neuroprotective Effects of Nicotinamide (Vitamin B_3_) on Neurodegeneration in Diabetic Rat Retinas

**DOI:** 10.3390/nu14061162

**Published:** 2022-03-10

**Authors:** Kyoung In Jung, Jeong-Sun Han, Chan Kee Park

**Affiliations:** Department of Ophthalmology, Seoul St. Mary’s Hospital, College of Medicine, The Catholic University of Korea, Seoul 06591, Korea; ezilean@hanmail.net (K.I.J.); winehan@catholic.ac.kr (J.-S.H.)

**Keywords:** apoptosis, diabetic retinopathy, DNA repair, nicotinamide, retinal ganglion cell, neuroprotection

## Abstract

The loss of inner retinal neurons is an initial event in diabetic retinopathy. In diabetic retinas, oxidative stress is increased, which could lead to increased oxidative DNA damage. Nicotinamide is a precursor to nicotinamide adenine dinucleotide, which contributes to the DNA damage response. We investigated whether nicotinamide plays a neuroprotective role in diabetic retinal neurodegeneration in terms of DNA repair. Male Sprague Dawley rats with streptozotocin-induced diabetes were orally administered nicotinamide (500 mg/kg/day) for 4 or 12 weeks. Oxidative stress exhibited by dihydroethidium was upregulated at 4 and 12 weeks after onset of diabetes, and nicotinamide treatment reduced oxidative stress at 4 weeks after induction of diabetes. Oxidative DNA damage measured by 8-hydroxy-2′-deoxyguanosine (8-OHdG) increased at 4 and 12 weeks after induction of diabetes and decreased following nicotinamide treatment. The elevated expression of glial fibrillary acidic protein (GFAP) induced by diabetes was attenuated by nicotinamide treatment. In Western blot analysis, the increased expression of cleaved PARP-1 in diabetes was attenuated by nicotinamide treatment at 12 weeks after induction of diabetes. The diabetes-induced apoptosis of inner retinal cells detected by the TUNEL assay was reduced by nicotinamide treatment. In conclusion, nicotinamide attenuated retinal neurodegeneration in diabetes, probably by reducing oxidative DNA damage and supporting DNA repair.

## 1. Introduction

Diabetic retinopathy is the major cause of blindness and leads to a reduction in the quality of life in patients with diabetes [1,2]. Growing evidence suggests that diabetic retinopathy is a complication due to retinal neurovascular units, and not just an instance of microvasculopathy [1,3,4]. Retinal neurodegeneration has been noted to develop early, before an overt microvascular change in patients with diabetes [5,6]. Oxidative stress, which is elevated in diabetic retinas, is regarded as one of the mechanisms of diabetic neurodegeneration [7,8,9], although the exact mechanism underlying inner retinal neurodegeneration in diabetes has not been clarified [9,10,11].

Reactive oxygen species can impair macromolecules such as DNA [12]. DNA damage induced by reactive oxygen species is the most commonly occurring damage in neuronal cells, with 10,000–100,000 oxidative lesions occurring on any given day in a typical mammalian cell [13,14,15]. Elevated oxidative DNA damage represented by modified, oxidized guanine bases (8-hydroxy-2′-deoxyguanosine, 8-OHdG) was found in diabetic retinas [16,17]. Moreover, the retina is easily affected by oxidative stress because it consumes a relatively greater amount of oxygen per unit weight of tissue compared to other tissue and is exposed to light directly [18]. Although the antioxidant itself could be helpful for suppressing oxidative stress, retinal damages produced by reactive oxygen species may be recovered by repair systems [19].

Following oxidative DNA damage, the DNA damage response is activated to repair DNA and enhance cell survival. Poly(ADP-ribose)polymerase (PARP) and nicotinamide adenine dinucleotide (NAD^+^) are some of the main molecules in DNA repair systems [20]. PARP-1 detects DNA breaks and plays a role in the polymerization of ADP-ribose units on target proteins using NAD^+^ as a substrate [20]. During mild pathologic stress, the activation of PARP-1 promotes DNA repair and cell survival [20]. Under severe oxidative stress, PARP activity is upregulated excessively, leading to a reduction in the NAD^+^ level [21]. Massive PARP-1 activation and subsequent NAD^+^ depletion can cause cell death [20,21,22]. One of the cell death mechanisms associated with relatively low NAD and PARP activation is apoptosis through PARP cleavage [23,24]. PARP was found to be increased in diabetic retinas [25,26]. A low NAD+ or NAD+/NADH ratio was observed in diabetic retinas [27,28]. The DNA damage response cannot function appropriately when there is not enough NAD^+^ for PARP to indicate the DNA sites in need of repair.

Given these findings, the administration of nicotinamide (a form of vitamin B_3_), a precursor to NAD^+^, might improve the DNA repair response in diabetic retinas. Nicotinamide decreased the cleavage of PARP-1 and gliosis in the retina of diabetic rats [25]. However, it has not been determined whether NAD+ can attenuate diabetic inner retinal neurodegeneration. Therefore, we investigated whether oral nicotinamide treatment had neuroprotective effects on diabetic retinal neurodegeneration regarding oxidative DNA damage and repair. 

## 2. Materials and Methods

### 2.1. Animals

Adult male Sprague Dawley rats (from seven to eight weeks, 200–300 g) were employed according to the Association for Research in Vision and Ophthalmology Statement on the Use of Animals in Ophthalmic and Vision Research. All animal research procedures were allowed by the Institutional Animal Care and Use Committee of the School of Medicine, The Catholic University of Korea Institutional Animal Care and Use Committee and the Department of Laboratory Animals, The Catholic University of Korea, Songeui Campus. 

### 2.2. Induction of Diabetes

Streptozotocin (STZ, Sigma-Aldrich, Saint Louis, MO, USA; 60 mg/kg bodyweight) in a 0.1 M citrate buffer solution (pH 4.5) was injected intraperitoneally to produce diabetes in rats. Similar-aged control rats were injected with the identical amount of citrate buffer solution. Serum glucose levels were determined employing an automated Accu-Check glucometer (Roche Diagnostics Ltd., Rotkreuz, Switzerland) at 3 days after injection of STZ. Rats with plasma glucose level greater than 350 mg/dL were determined to be diabetic and adopted for further test. Body weight and plasma glucose level were checked weekly after onset of diabetes.

### 2.3. Allocation of Groups and Drug Treatments

**Figure 1:**
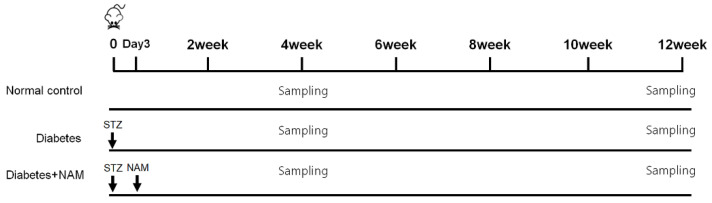
Timetable of the experimental design. Serum glucose values were assessed at 3 days after induction of diabetes. From the day when diabetes was confirmed, the rats in the nicotinamide group were given 500 mg/kg/day oral administration of nicotinamide in drinking water for 4 weeks or 12 weeks. NAM, nicotinamide; STZ, streptozotocin.

From the day diabetes was confirmed, the rats in the nicotinamide group were given 500 mg/kg/day of nicotinamide in drinking water for 4 or 12 weeks (Figure 1) [29]. The control group was given drinking water without drug. Twelve rats were randomly allocated to each group. 

### 2.4. Immunofluorescence Staining

Enucleation of eyeball was carried out after sacrificing rats by CO_2_ inhalation. Enucleated eyes were washed with phosphate-buffered saline (PBS). Eyeball was immersed in 4% paraformaldehyde in 0.1 M phosphate buffer for 20 min. Following extracting the cornea and the lens, the posterior eyecups were immersed in 4% paraformaldehyde in 0.1 M phosphate buffer for one hour at 4 °C. The posterior eyecups were rinsed with PBS and immersed in 0.1 M phosphate buffer, including 25% sucrose, at 4 °C overnight. After rinsing with PBS, the samples were put in optimal cutting temperature compound and frozen with liquid nitrogen. Cryostat sections (12 µm) were made and kept at −20 °C.

**Figure 2:**
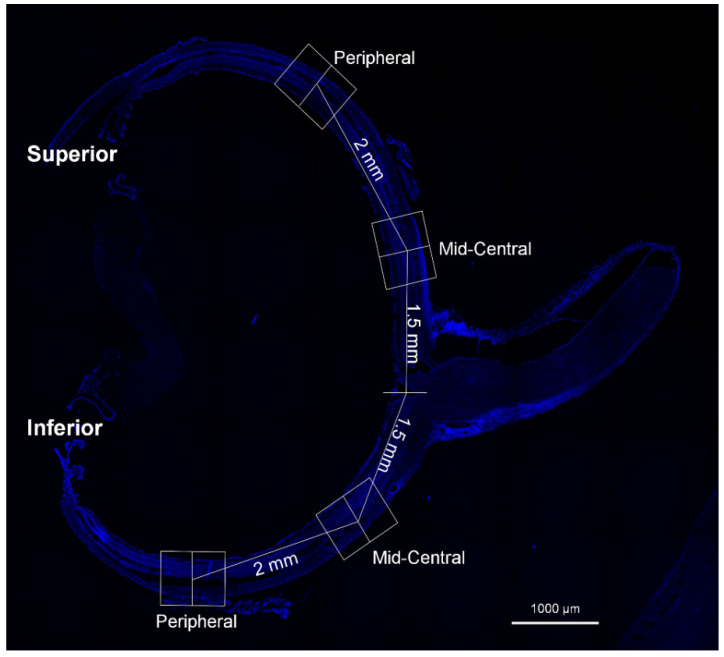
Representative posterior eyecup image stained with DAPI for analysis. Each eyecup was split into 2 mid-central (1.5 mm from the optic nerve) and 2 peripheral regions (3.5 mm from the optic nerve) for image assay.

Cryosections were thawed, air-dried, and rinsed with PBS. They were treated with 3% Triton X-100 for 30 min and put with 10% normal donkey serum for one hour. The slides were treated with anti-glial fibrillary acidic protein (GFAP; Millipore, MA, USA, #MAB360; 1:400), 8-OHdG (Abcam, Cambridge, UK, #ab62623; 1:500), PARP-1 (Santa Cruz Biotechnology, Dallas, TX, USA, #sc-74470; 1:200), and anti-gamma H2AX (phosphoS139; Abcam, Cambridge, UK, #ab81299; 1:200) overnight at 4 °C. The sections were then incubated with Alexa Fluor 488-labeled goat anti-mouse IgG (Thermo Fisher Scientific, Waltham, MA, USA, #A-11001) or Alexa Fluor 546-labeled goat anti-rabbit IgG (Thermo Fisher Scientific, Waltham, MA, USA, #A-11010) for 1 h. The sections were mounted using VECTASHIELD mounting medium with DAPI (Vector Laboratories, Burlingame, CA, USA). Image was analyzed after each eyecup was divided into 2 mid-central (approximately 1.5 mm from the optic nerve) and 2 peripheral (approximately 3.5 mm from the optic nerve) areas (Figure 2). Images were analyzed using Image J software (version 1.40; National Institute of Health, Bethesda, MD, USA). Multi-color images are divided into separate channels, which were changed to grayscale before processing. We assessed the fluorescence level higher than a threshold using the “set measurements” tool.

### 2.5. Measurement of Reactive Oxygen Species

Retinal sections were incubated with 5 µM dihydroethidium (DHE; Invitrogen Waltham, MA, USA, #D11347) for 20 min as the manufacturer recommended. DHE is converted to an ethidium derivative when it reacts with intracellular superoxide [30]. The ethidium derivative binds to the deoxyribonucleic acid, thereby letting the cell emit red fluorescence [31]. The samples were photographed at an excitation wavelength of 520 nm and an emission wavelength of 610 nm. 

### 2.6. Terminal Deoxynucleotidyl Transferase-Mediated dUTP Nick-End Labeling

To evaluate apoptotic cells, we conducted a terminal deoxynucleotidyl transferase-mediated dUTP nick-end labeling (TUNEL) assay according to the manufacturer’s manuals (In Situ Cell Detection Kit, Roche, Rotkreuz, Switzerland). Regarding double-labeled staining, the sections were treated with mouse anti-NeuN antibody (NeuN; Millipore, MA, USA, #MAB377; 1:200), followed by incubation with Alexa Fluor 488-labeled goat anti-mouse IgG (Thermo Fisher Scientific, Waltham, MA, USA, #A-11001) for one hour. The percentage of TUNEL-positive cells was calculated after the number of TUNEL-positive cells was divided by the number of DAPI-positive cells in the retinal ganglion cell layer (GCL) and then multiplied by 100. 

### 2.7. Western Blot Analysis 

Protein extraction and the Western blotting were performed as previously stated [32]. Retinal tissues were lysed in RIPA buffer, and the total protein value was analyzed using a standard bicinchoninic acid assay (Pierce). Sample buffer was supplemented to retinal tissue, including 30 μg of total protein. The protein was isolated utilizing 10% SDS-PAGE and fixed onto a nitrocellulose membrane. The membranes were rinsed and treated with 5% skim milk in Tris-buffered saline/Tween 20 (TBST) buffer for 1 h at room temperature. Then, the membranes were put with antibodies with regard to PARP-1 (Cell Signaling, Danvers, MA, USA, #9542; 1:1000), cleaved PARP-1 (Abcam, Cambridge, UK, #ab32064; 1:1000), and actin (Santa Cruz Biotechnology, Dallas, TX, USA, #sc-47778; 1:200) overnight at 4 °C. The membranes were immersed in the TBST buffer containing 5% skim milk and a horseradish peroxidase-conjugated goat anti-rabbit or goat anti-mouse IgG as the secondary antibody for 1 h. Proteins were detected by ECL Western blotting substrate (Thermo Scientific, Waltham, MA, USA), and immunoblot bands were checked by an image analyzer system (Syngene, Cambridge, UK). Quantification was performed utilizing ImageJ software (NIH, Bethesda, MD, USA). 

### 2.8. Data Analysis 

Statistical analyses were carried out using SPSS software (ver. 17.0; SPSS Inc., Chicago, IL, USA). All data are indicated as the mean ± SD. Multiple comparisons among the groups were made using Kruskal–Wallis and post hoc Dunn’s test. The results with *p*< 0.05 were considered statistically significant.

## 3. Results

### 3.1. Body Weight and Blood Glucose

**Figure 3:**
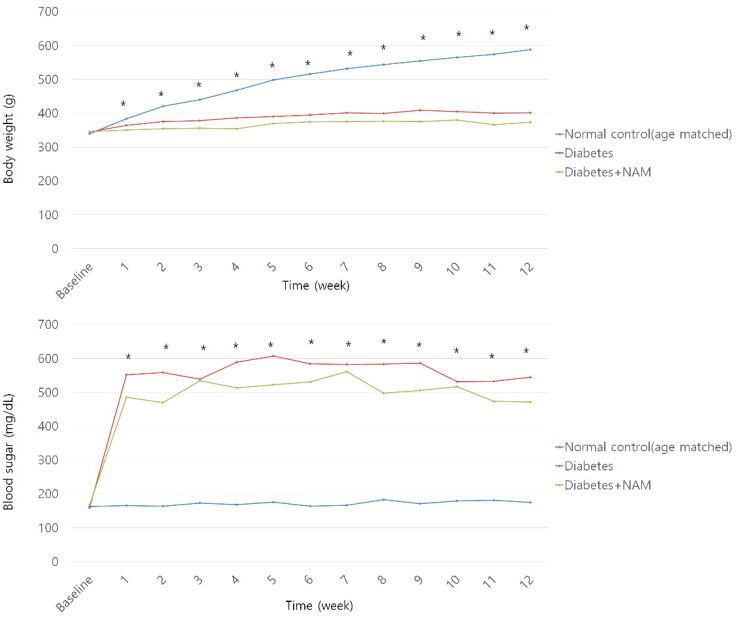
Body weight and blood glucose level. Body weight was lighter in the diabetes group than in the normal control group from one week to twelve weeks after streptozotocin injection, regardless of drug treatment (all *p* < 0.05). Blood glucose was elevated in the diabetic rats compared to the normal control rats from one week to twelve weeks after onset of diabetes (all *p* < 0.05). NAM, nicotinamide. *, significant difference exists between groups (*p* < 0.05).

The body weight was lighter in the diabetes group, regardless of drug treatment, than in the normal control group from one to twelve weeks after STZ injection (all *p* < 0.05, Figure 3). The diabetes group showed higher serum glucose levels than in the normal control group from one to twelve weeks after STZ injection (all *p* < 0.05).

### 3.2. Oxidative Stress and Oxidative DNA Damage 

**Figure 4:**
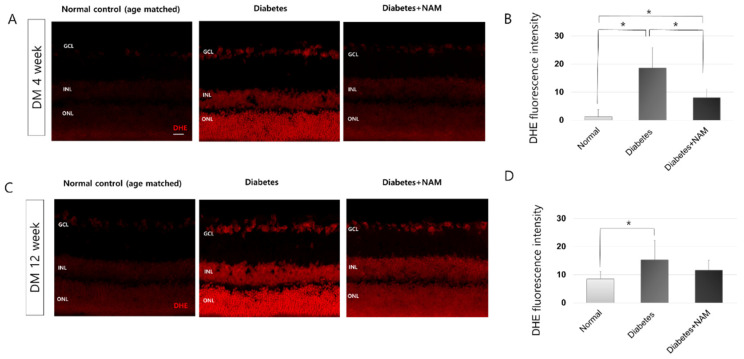
Dihydroethidium (DHE) staining, which detects superoxide (**A**–**D**). Four and twelve weeks after induction of diabetes, retinas showed extensively increased staining for DHE spanning the vertical retinal section (both *p* < 0.001). Nicotinamide treatment reduced DHE expression at 4 weeks after onset of diabetes (*p* < 0.001). DHE, dihydroethidium; DM, diabetes mellitus; NAM, nicotinamide. Scale bar = 20 μm. GCL, ganglion cell layer. INL, inner nuclear layer. ONL, outer nuclear layer. *, significantly different as indicated (*p* < 0.05).

DHE staining, which detects superoxide, was performed to identify the degree of oxidative stress in diabetic retinas (Figure 4). Four and twelve weeks after the induction of diabetes, retinas revealed stronger labeling for DHE in all layers than the normal control retinas (Both *p* < 0.001). Diabetes leads to increased oxidative stress in the retina. Nicotinamide treatment decreased DHE expression at 4 weeks after induction of diabetes (*p* < 0.001). At 12 weeks after induction of diabetes, nicotinamide reduced DHE staining induced by diabetes, but the difference was not statistically significant (*p* = 0.587). 

**Figure 5:**
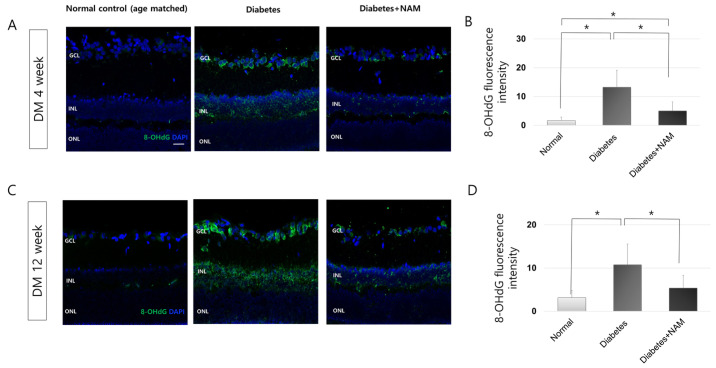
Oxidative DNA damage. 8-Hydroxy-2′-deoxyguanosine (OHdG) expression was observed in the ganglion cell layer (GCL) at 4 weeks after injection of streptozotocin. 8-OHdG immunostaining was extended to the inner nuclear layer at 12 weeks after onset of diabetes (**A**,**C**). Nicotinamide treatment attenuated increased 8-OHdG labeling by diabetes at 4 and 12 weeks after induction of diabetes (*p* = 0.002, 0.005, respectively; (**B**,**D**)). Scale bar = 20 μm. DM, diabetes mellitus; NAM, nicotinamide. GCL, ganglion cell layer. INL, inner nuclear layer. ONL, outer nuclear layer. *, significantly different as indicated (*p* < 0.05).

To identify the oxidative DNA damage level in diabetic retinas, we performed 8-OHdG immunofluorescence staining (Figure 5). 8-OHdG expression was observed in the GCL at 4 weeks after STZ injection, and 8-OHdG labeling was increased and extended to the inner nuclear layer at 12 weeks after induction of diabetes. Compared to the control group both at 4 and 12 weeks after induction of diabetes, 8-OHdG expression was higher in the diabetic group (both *p* < 0.001). Moreover, nicotinamide treatment reduced the 8-OHdG immunofluorescence staining level at 4 and 12 weeks after induction of diabetes (*p* = 0.002, 0.005, respectively). 

### 3.3. Glial Activation in the Retina

**Figure 6:**
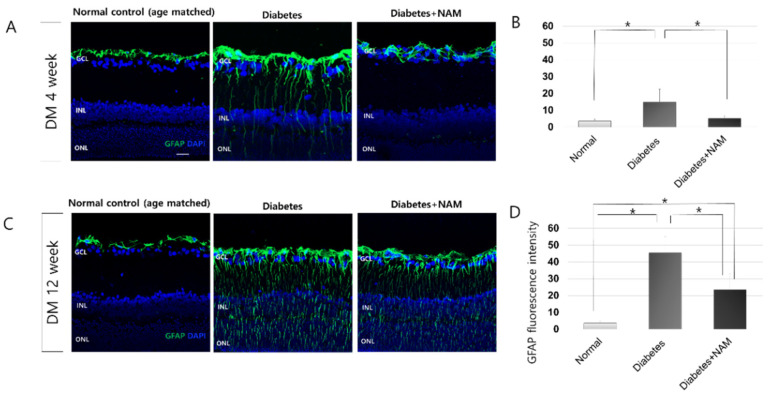
Glial activation. Glial fibrillary acidic protein (GFAP) immunofluorescence staining was increased, extending to the outer nuclear layer compared to the normal control group at 4 and 12 weeks after onset of diabetes (**A**,**C**). Nicotinamide treatment significantly attenuated GFAP expression at 4 and 12 weeks after induction of diabetes (Both *p* < 0.001; (**B**,**D**)). Scale bar = 20 μm. DM, diabetes mellitus; NAM, nicotinamide. GCL, ganglion cell layer. INL, inner nuclear layer. ONL, outer nuclear layer. *, significantly different as indicated (*p* < 0.05).

In the normal control group, GFAP expression was confined to astrocytes and the end feet of Müller cells at the inner limiting membrane in vertical retinal sections (Figure 6). At 4 weeks after injection of STZ, GFAP immunofluorescence was increased, spreading to the outer nuclear layer compared to the normal control group; this trend continued at 12 weeks after induction of diabetes (both *p* < 0.001). Nicotinamide treatment significantly attenuated GFAP immunofluorescence staining at 4 and 12 weeks after induction of diabetes (Both *p* < 0.001). 

### 3.4. PARP-1 

**Figure 7:**
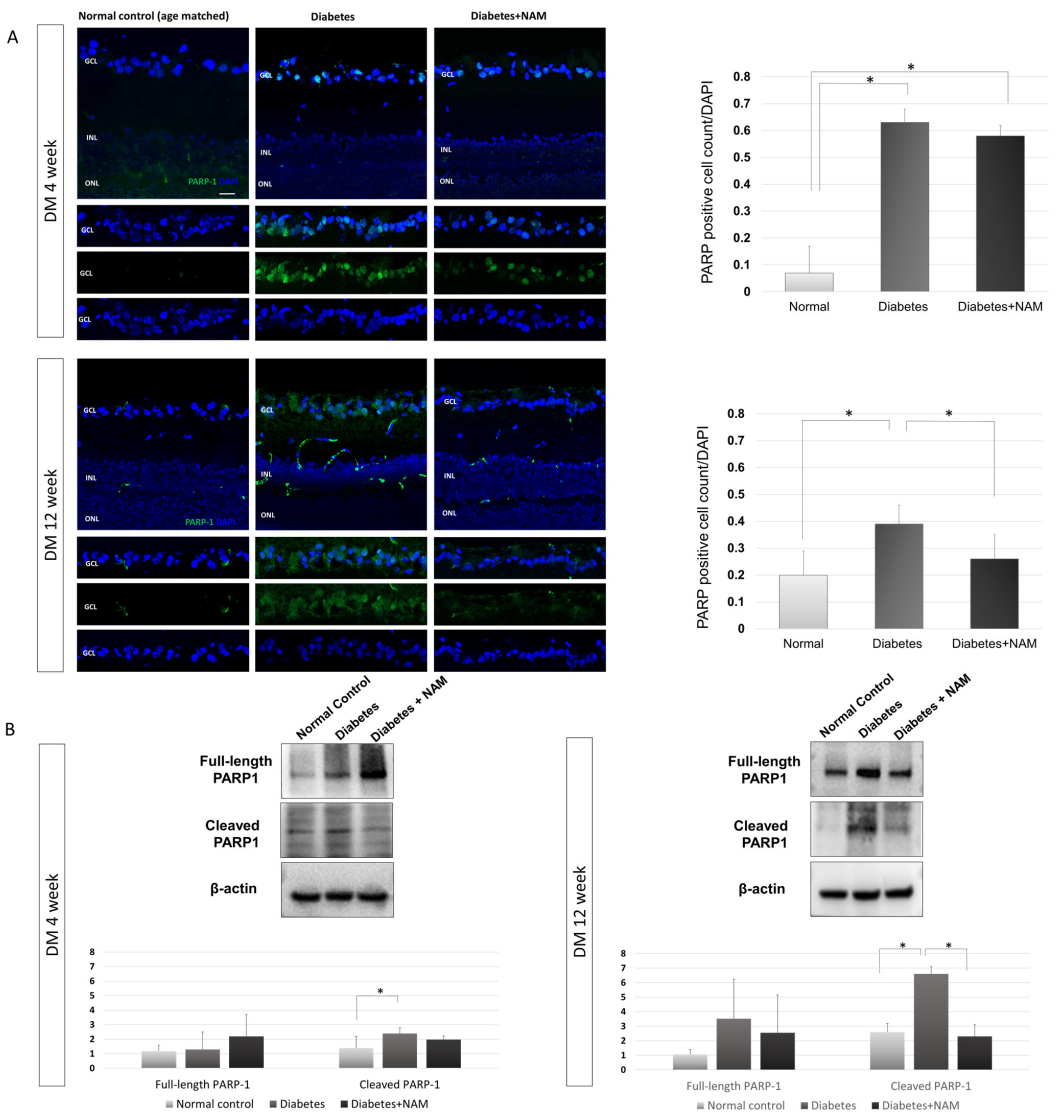
(**A**) Poly(ADP-ribose)polymerase (PARP)-1 immunofluorescence staining. The proportion of PARP-1-positive cells was elevated in the diabetes group compared to the normal control group at 4 weeks and 12 weeks after onset of diabetes (both *p* < 0.001). An increased expression of PARP-1 in diabetic conditions was decreased by nicotinamide treatment at 12 weeks after induction of diabetes (*p* < 0.001). (**B**) Western blotting of poly(ADP-ribose)polymerase (PARP)-1. There was no significant difference in the expression of full-length PARP-1 according to the presence of diabetes or nicotinamide treatment at 4 or 12 weeks after onset of diabetes (*p* = 0.228, *p* = 0.103, respectively) Cleaved PARP-1 expression was elevated in the diabetes group compared to the normal control group at 4 and 12 weeks after induction of diabetes (*p* = 0.002, *p* < 0.001, respectively). Elevated expression of cleaved PARP-1 was reduced by nicotinamide treatment 12 weeks after onset of diabetes (*p* < 0.001). Scale bar = 20 μm. DM, diabetes mellitus; NAM, nicotinamide. GCL, ganglion cell layer. INL, inner nuclear layer. ONL, outer nuclear layer. *, significantly different as indicated (*p* < 0.05).

PARP-1 immunofluorescence staining was scant in the normal control retinas (Figure 7). At 4 and 12 weeks after induction of diabetes, PARP-1 immunostaining was found mostly in the GCL. The proportion of PARP-1-positive cells was increased in the diabetes group compared to the normal control group at 4 weeks and 12 weeks after induction of diabetes (Both *p* < 0.001). The upregulated expression of PARP-1 by diabetes was attenuated by nicotinamide treatment at 12 weeks after induction of diabetes (*p* < 0.001).

As indicated by the Western blot analysis, there was no significant difference in the expression of full-length PARP-1 among the normal control group, diabetes group, and diabetes with nicotinamide treatment group at 4 or 12 weeks after induction of diabetes (*p* = 0.228, *p* = 0.103, respectively; Figure 7). However, cleaved PARP-1 expression was increased in the diabetes group compared to the normal control group at 4 and 12 weeks after induction of diabetes (*p* = 0.002, *p* < 0.001, respectively). An elevated expression of cleaved PARP-1 was attenuated by nicotinamide treatment at 12 weeks after the onset of diabetes (*p* < 0.001). 

### 3.5. Apoptotic Retinal Ganglion Cell Death

**Figure 8:**
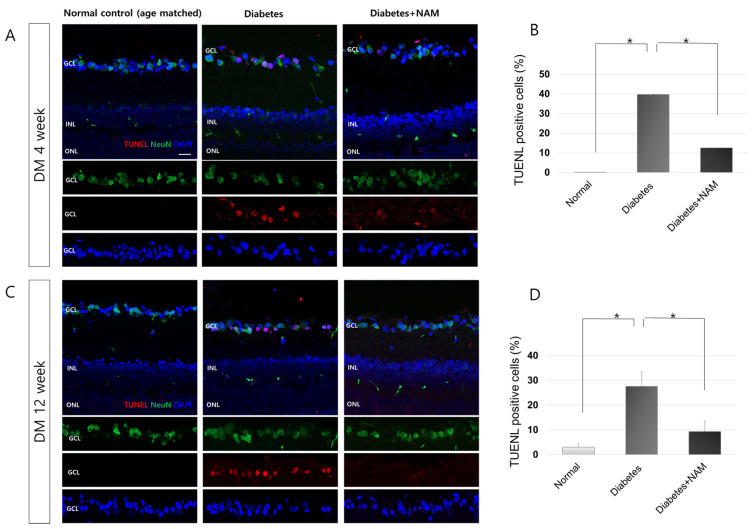
Apoptosis in the diabetic retina. Terminal deoxynucleotidyl transferase-mediated dUTP nick-end labeling (TUNEL)-positive cells were observed in the ganglion cell layer (GCL) and colocalized with anti-NeuN antibody at 4 and 12 weeks after induction of diabetes (**A**,**C**). The percentage of TUNEL-positive cells was higher in diabetic retinas than in the normal control (*p* = 0.002, *p* < 0.001) at 4 and 12 weeks, respectively, after onset of diabetes (**B**,**D**). The proportion of apoptotic cells in diabetic retinas was reduced by administration of nicotinamide at 4 and 12 weeks after induction of diabetes (*p* = 0.002, *p* < 0.001, respectively). Scale bar = 20 μm. DM, diabetes mellitus; NAM, nicotinamide. INL, inner nuclear layer. ONL, outer nuclear layer. *, significantly different as indicated (*p* < 0.05).

The normal control retinas did not display TUNEL-positive cells, which were, however, found in the GCL 4 and 12 weeks after onset of diabetes (Figure 8). TUNEL-positive cells were mostly colocalized with an anti-NeuN antibody, which indicates retinal ganglion cells (RGCs) in the diabetic retina. The proportion of TUNEL-positive cells was greater in the diabetes group than in the normal control group (*p* = 0.002 and *p* < 0.001 at 4 and 12 weeks after induction of diabetes, respectively). The application of nicotinamide lowered the proportion of apoptotic cells in the diabetic retinas at 4 and 12 weeks after induction of diabetes (*p* = 0.002, *p* < 0.001, respectively).

### 3.6. Ultrastructural Features of the Optic Nerve 

**Figure 9:**
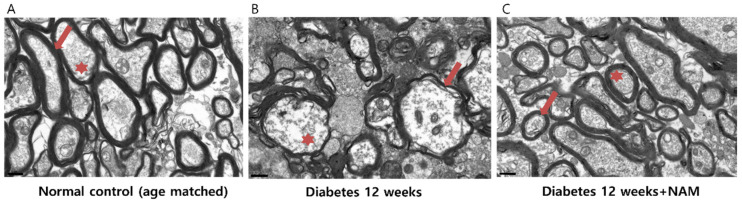
Electron microscopy of cross-sections through the region surrounding the distal myelinated optic nerve demonstrated that axons with normal axoplasm were surrounded by myelin sheaths in the normal control eyes (**A**). In the diabetic retinas, myelin sheaths were thinned and unorganized, and axoplasm was sparse compared to the control retinas (**B**). In the retinas of diabetes model mice that underwent nicotinamide treatment, myelin sheaths and axoplasm were relatively preserved (**C**). Scale bars = 0.5 μm. NAM, nicotinamide. Red asterisk, axoplasm. Red arrow, myelin sheaths.

Electron microscopy of cross-sections through the region surrounding the distal myelinated optic nerve showed healthy axons with normal axoplasm surrounded by myelin sheaths in the normal control group (Figure 9). In the diabetic retinas, myelin sheaths were thinned and unorganized, and the axoplasm was sparse compared to the control retinas. In the retinas of the diabetes model mice treated with nicotinamide, myelin sheaths and axoplasm were relatively conserved.

## 4. Discussion

We demonstrated that gliosis, oxidative stress, and oxidative DNA damage increased at 4 and 12 weeks after induction of diabetes in rats. Gliosis and oxidative DNA damage were reduced following nicotinamide treatment, a precursor to NAD^+^. The elevated expression of cleaved PARP-1 caused by diabetes was decreased following nicotinamide application. Nicotinamide treatment attenuated the loss of retinal ganglion cells induced by diabetes. Nicotinamide seemed to attenuate inner retinal neurodegeneration by enhancing DNA repair and decreasing the cleavage of PARP-1. 

In terms of oxidative stress, DHE staining, which detects superoxide, was increased in all retinal layers in the diabetes group at 4 and 12 weeks after induction of diabetes (Both *p* < 0.001). Sasaki et al. reported that DHE staining was elevated, with the expression spanning the entire retina in diabetic mice [30], as was found in this study. The acceleration of glycation and the mitochondrial electron transport system, which reaches the maximum threshold in hyperglycemic conditions, seemed to contribute to elevated oxidative stress in diabetic retinopathy [33,34].

Oxidative DNA lesions increased in the inner retina, as indicated by 8-OHdG expression, the most common oxidative DNA lesion [35], especially in the GCL at 4 and 12 weeks after induction of diabetes (both *p* < 0.001). Dong et al. also found that retinal 8-OHdG staining was elevated in diabetic *db/db* mice [16]. Oxidative DNA damage was alleviated by a treatment of nicotinamide both 4 and 12 weeks after STZ injection (*p* = 0.002, 0.005, respectively). NAD^+^ has been found to reduce oxidative DNA damage in primary melanocytes and in primary cortical neurons [36,37], which is in accordance with this study. NAD^+^ is a cofactor in cellular metabolism and plays a critical role in DNA repair [38]. There is a possibility that nicotinamide treatment reduced oxidative DNA damage by lowering oxidative stress or improving DNA repair. The increased DHE reaction in diabetic conditions did not decrease significantly following nicotinamide application at 12 weeks after induction of diabetes. Therefore, we assumed that the reduction in oxidative DNA damage by NAD^+^ seemed to mainly be attributed to the enhancement of DNA repair. 

GFAP expression was elevated in diabetic retinas at 4 and 12 weeks after diabetes onset (both *p* < 0.001). We previously reported elevated GFAP, which is considered a sensitive non-specific marker of retinal stress or injury, [39] in diabetic rat retinas [32,40]. Increased GFAP indicates reactive gliosis and might result from hyperglycemia, oxidative stress, hypoxia, or inflammation in diabetic retinas [41,42]. Nicotinamide treatment reduced reactive gliosis in diabetic retinas at 4 and 12 weeks after induction of diabetes (both *p* < 0.001) in accordance with Guzyk’s study showing that nicotinamide treatment decreased retinal GFAP expression in the retina at 8 weeks after diabetes onset [25]. Early glial activation could protect retinal tissue, but chronic gliosis could be detrimental by aggravating retinal neurodegeneration [43]. In this study, RGC apoptosis was increased after onset of diabetes and was reduced by nicotinamide treatment. Therefore, it is possible that nicotinamide administration attenuated the detrimental effects of reactive gliosis on diabetic retinas. The suppression of reactive gliosis by nicotinamide treatment was beneficial in traumatic brain injury animal models [44,45]. The reduction in oxidative DNA damage by nicotinamide might contribute to diminishing gliosis in diabetic retinopathy [27].

PARP activity is performed primarily by PARP-1 (85–90%) and secondarily by PARP-2 (10–15%) [46]. Therefore, we evaluated PARP-1 expression by immunofluorescence and Western blot analysis. The expression of PARP-1, measured by immunofluorescence staining, was increased in the diabetes group, and nicotinamide treatment alleviated PARP-1 immunofluorescence. The pattern of PARP-1 expression seemed to be contributed mainly by cleaved PARP-1, with reference to the results of Western blotting. In the Western blot analysis, only cleaved PARP-1 expression increased at 4 and 12 weeks after the onset of diabetes (*p* = 0.002, *p* < 0.001, respectively); full-length PARP-1 expression did not show a significant difference between groups. Elevated cleaved PARP-1 expression was suppressed by nicotinamide treatment at 12 weeks after induction of diabetes (*p* < 0.001). This result is in accordance with a previous study showing that upregulated cleaved PAPR-1 expression in diabetic retinas was downregulated by the administration of nicotinamide [25].

Retinal cell apoptosis analyzed by TUNEL staining was increased at 4 and 12 weeks after onset of diabetes (*p* = 0.002, *p* < 0.001, respectively) and decreased with nicotinamide treatment (*p* = 0.002, *p* < 0.001 at 4 and 12 weeks, respectively). Apoptotic cells were primarily located in the GCL, and most of the TUNEL-positive cells were colocalized with anti-NeuN antibodies. These findings indicate that most apoptotic cells were RGCs in diabetic retinas. Many studies, including our group’s previous study, reported early inner retinal neurodegeneration in diabetic animal models and in diabetic patients [3,5,6,9,32]. RGCs have been found to be greatly susceptible to hypoxic stress or the neurodegenerative pathology in diabetes [9,11,47].

Nicotinamide treatment, a precursor of NAD^+^, decreased inner retinal neurodegeneration in diabetic retinas. To our knowledge, there have been no reports investigating the effects of nicotinamide on retinal neurodegeneration in diabetic retinas, even though Guzyk reported that nicotinamide decreased gliosis and cleaved PARP-1 expression [25].

**Figure 10:**
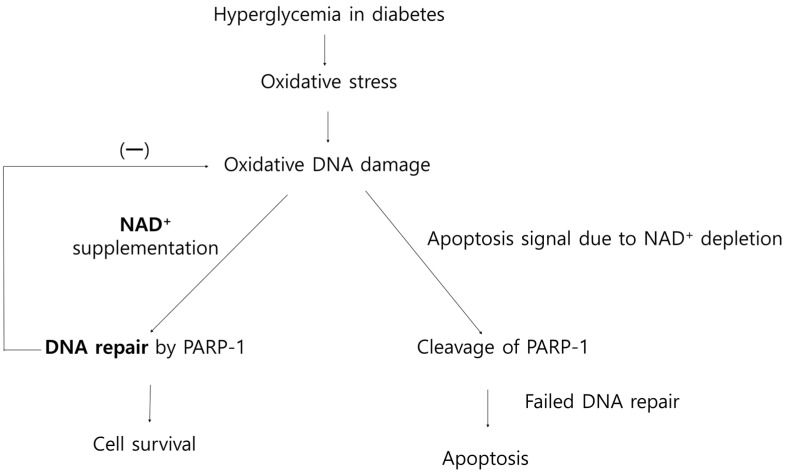
Hypothetical overview of oxidative DNA damage, DNA repair, and effects of nicotinamide (precursor of NAD^+^) supplementation on retinal neurodegeneration.

NAD^+^ is required for PARP-1 to indicate damaged regions of DNA by catalyzing the formation of long poly(ADP-ribose) (PAR) polymers on target proteins as a substrate. PAR plays a role as a platform to recruit DNA repair proteins to the damaged area [20,48]. In this study, increased oxidative stress, oxidative DNA damage, and PARP-1 activation were observed in diabetic retinas. The activation of PARP-1 consumes 80–90% of NAD^+^ from its steady-state value in minutes after DNA damage, and, with each catalytic process, PARP-1 spends up to 200 molecules of NAD^+^ [49,50]. In the face of substantial DNA damage, such as severe oxidative stress, PARP could be inordinately activated and lead to subsequent, massive NAD^+^ consumption [20]. NAD^+^ exhaustion leads to ATP depletion because ATP is used during NAD^+^ biosynthesis, and NAD is required for ATP production in the process of oxidative phosphorylation [50,51]. At lower levels of NAD/ATP, PARP-1 is cleaved by caspase-3 and -7 [24,52]. Cleaved PARP-1 loses its catalytic activity for DNA repair [24,53] and leads to apoptosis through caspase-mediated or independent DNA fragmentation [52,54]. In this study, cleaved PARP-1 expression was increased in diabetic retinas and decreased by the administration of nicotinamide. Given these findings, we assumed that a sufficient nicotinamide supply diminished the overactivation of PARP-1 and subsequent cleavage of PARP-1. The suppression of PARP-1 overactivation by the NAD^+^ supply through nicotinamide seemed to reduce oxidative DNA damage and ultimately attenuated RGC apoptosis by improving DNA repair (Figure 10). 

In this study, the treatment of nicotinamide started from the day when diabetes was confirmed. Pretreatment with a high dose of nicotinamide has been found to be effective in preventing the onset of diabetes in streptozotocin-injected animals [27,55]. Fan et al. suggested that the mechanisms of NAD dysregulation overlap with those of diabetic complications [27]. Therefore, the prophylactic treatment of nicotinamide prior to onset of diabetes might be more effective than the treatment of nicotinamide as an intervention after onset of diabetes. However, The European Nicotinamide Diabetes Intervention Trial failed to prove the suppressive effects of nicotinamide for the development of type 1 diabetes [55]. Further studies should be performed to determine the appropriate time for the treatment of nicotinamide. 

Diabetic retinopathy is characterized by early RGC loss, which is the main feature of glaucoma, although two diseases have a dissimilar pathogenesis [5,9,32,56]. We previously found that a low intake of vitamin B_3_ was associated with a higher probability of having glaucoma [57]. Several reports also demonstrated that the oral treatment of nicotinamide revealed the neuroprotective effects on retinal ganglion cells in glaucoma animal models or glaucoma patients [29,58]. The randomized clinical trial showed that the administration of a dose of 1.5 g/day followed by 3.0 g/day was effective in the improvement of the inner retinal function in patients with glaucoma [58]. Nicotinamide is the amide form of vitamin B3 and is a widely available supplement. High-dose nicotinamide (no more than 3 g/day) has shown a relatively good tolerability and minimal adverse effects, such as skin flushing and nausea (≤1.5%) [59], and fewer than high-dose niacin [59,60]. One report showed that hepatotoxicity was found in one patient taking 9 g/day of nicotinamide [61]. Experimental studies showing the neuroprotective effects of nicotinamide on diabetic retina or the possibility of that [25,29,37,58] could justify performing randomized clinical trials using an appropriate dose of nicotinamide in diabetic patients. 

In conclusion, oral nicotinamide supplementation could have neuroprotective potential against diabetic inner retinal neurodegeneration, even though further clinical studies are needed to confirm the effects of nicotinamide in diabetic retinopathy. 

## Data Availability

Not applicable.
